# Directed Chemical Evolution with an Outsized Genetic Code

**DOI:** 10.1371/journal.pone.0154765

**Published:** 2016-08-10

**Authors:** Casey J. Krusemark, Nicolas P. Tilmans, Patrick O. Brown, Pehr B. Harbury

**Affiliations:** 1 Department of Biochemistry, Stanford University, Stanford, California, United States of America; 2 Department of Medicinal Chemistry and Molecular Pharmacology, Purdue University, West Lafayette, Indiana, United States of America; Imperial College London, UNITED KINGDOM

## Abstract

The first demonstration that macromolecules could be evolved in a test tube was reported twenty-five years ago. That breakthrough meant that billions of years of chance discovery and refinement could be compressed into a few weeks, and provided a powerful tool that now dominates all aspects of protein engineering. A challenge has been to extend this scientific advance into synthetic chemical space: to enable the directed evolution of abiotic molecules. The problem has been tackled in many ways. These include expanding the natural genetic code to include unnatural amino acids, engineering polyketide and polypeptide synthases to produce novel products, and tagging combinatorial chemistry libraries with DNA. Importantly, there is still no small-molecule analog of directed protein evolution, i.e. a substantiated approach for optimizing complex (≥ 10^9 diversity) populations of synthetic small molecules over successive generations. We present a key advance towards this goal: a tool for genetically-programmed synthesis of small-molecule libraries from large chemical alphabets. The approach accommodates alphabets that are one to two orders of magnitude larger than any in Nature, and facilitates evolution within the chemical spaces they create. This is critical for small molecules, which are built up from numerous and highly varied chemical fragments. We report a proof-of-concept chemical evolution experiment utilizing an outsized genetic code, and demonstrate that fitness traits can be passed from an initial small-molecule population through to the great-grandchildren of that population. The results establish the practical feasibility of engineering synthetic small molecules through accelerated evolution.

## Introduction

Evolution, the change in the inherited characteristics of biological populations over successive generations, accounts for the diversity of life on earth. Thirty thousand years ago humans began to harness it as a tool for the selective breeding of animals and crops. Today, evolution is routinely used to engineer macromolecules [1–4]. The enabling technology involves the creation of a microscopic ecosystem populated by autonomous DNA, RNA or protein units. Each member of this population is linked to its own genetic blueprint. Subjecting the population to a selective pressure enriches for variants with a particular functional property, for example the ability to bind to a pre-determined molecular target. The genetic blueprints of the survivors are amplified, diversified by mutation or recombination, and then used to program the biosynthesis of a child generation [5]. After cycles of selection, gene amplification and resynthesis, molecular species with exceptional binding or catalytic properties emerge from the population. This selective breeding of macromolecules has provided an important experimental model for natural evolution [6,7]. The technology now dominates industrial protein engineering, and has made fundamental contributions in green-tech, agriculture and protein therapeutics [8–10].

Extending directed evolution further into the molecular realm, to the products of synthetic organic chemistry, could fundamentally change our exploration and understanding of chemical space. There has been significant progress in this direction based on the concept of DNA-tagged small-molecule libraries, which are assembled from a large building-block alphabet by combinatorial chemistry [11–16]. Each compound is covalently linked to a unique DNA tag that records its synthetic history. Following enrichment for molecules with a desired property, the DNA tags are sequenced to identify candidate hits. DNA-tagged approaches have been applied to small-molecule collections with complexities in the 10^9^ range, yielding protein ligands with sub-nanomolar dissociation constants in some cases [15,17,18].

Although DNA-tagged library techniques mimic many aspects of natural evolution, they lack its most fundamental property: the inheritance of advantageous characteristics over successive generations. The DNA tags do not act as genes. They provide only a retrospective record of how a molecule was synthesized. As a result, there is no way to reproduce (resynthesize) a small-molecule library starting from a pool of enriched DNA tags. This has important consequences. Chemical diversity is limited to the distinct molecules that can be sampled in a single library generation. It is not possible to follow an evolutionary trajectory though a complex chemical space, because recombination and mutation of advantageous genes cannot occur. Moreover, there is no exponential enrichment of desirable traits over multiple generations.

**Fig 1:**
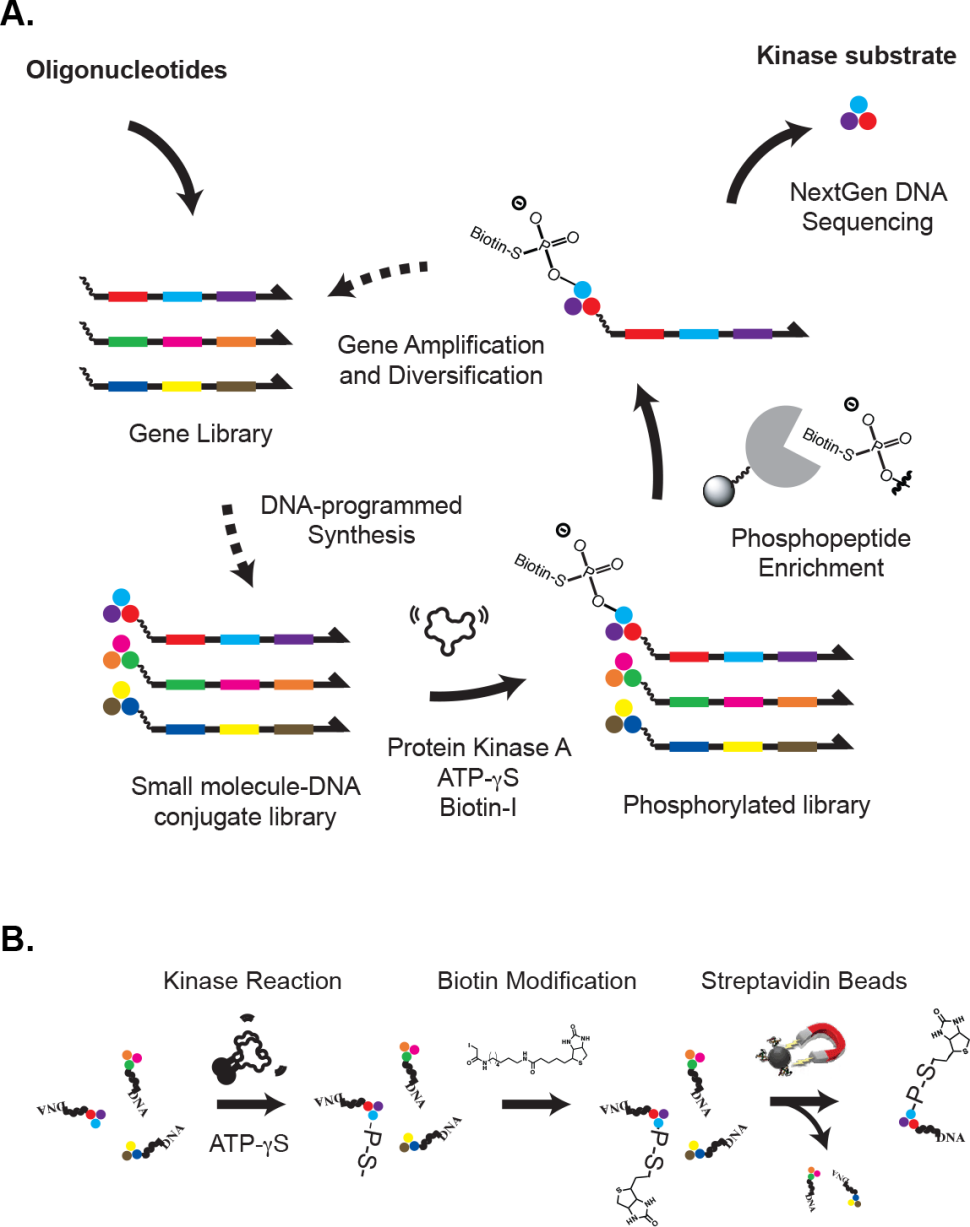
**A. Directed evolution of kinase substrates.** An initial population of DNA genes was chemically translated into peptide-DNA conjugates using a DNA-programmed combinatorial library synthesis. The library of peptide-DNA conjugate was treated with protein kinase A, and phosphorylated conjugates were isolated. The genes associated with the phosphorylated conjugates were then amplified by the polymerase chain reaction, diversified by recombination, and used to program the synthesis of a subsequent library generation. After generations 2–4, the gene population was sequenced. Peptides encoded by enriched genes were synthesized individually without DNA, and tested for their ability to function as protein kinase A substrates. The gene amplification, diversification and DNA-programmed library synthesis steps (dashed arrows) were required to close the evolutionary cycle. DNA-tagged library techniques utilize a linear process, consisting of an unprogrammed synthesis of small molecule-DNA conjugates, a selection for function, and DNA sequencing (e.g. solid arrows). **B. Selection for kinase substrates.** The peptide-DNA conjugate library was incubated with protein kinase A and ATP-γ-S. The enzymatically treated library was then alkylated with biotin iodoacetamide, which coupled a biotin moiety to thiophosphorylated peptides. The biotinylated peptide-DNA conjugates were affinity purified on paramagnetic streptavidin beads. See S1 Fig for a quantification of phosphopeptide enrichment.

**Fig 2:**
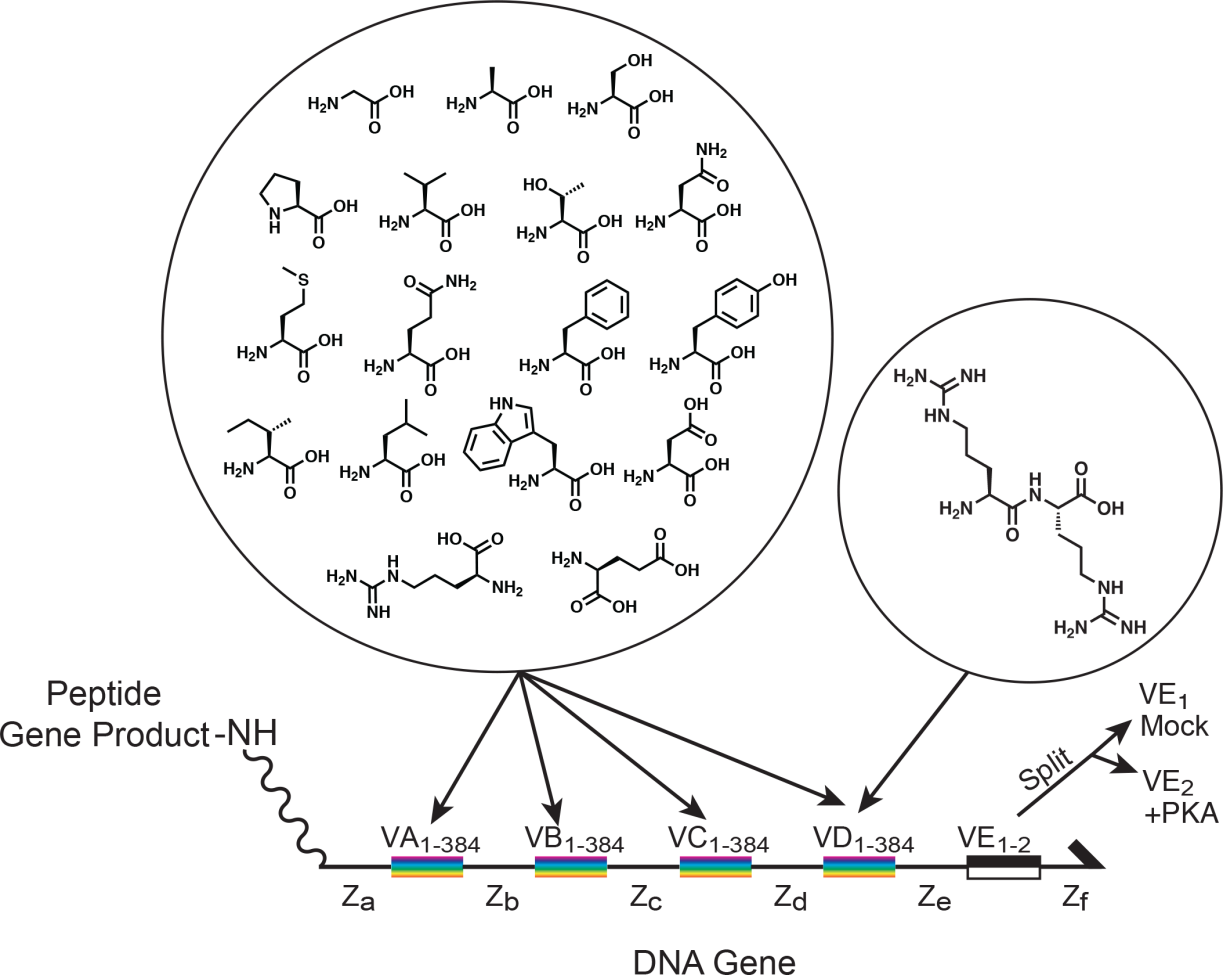
Gene structure. The genes that programed the synthesis of specific tetrapeptides were made up of four amino-acid coding regions (VA-VD, rainbow bars). 384 distinct DNA codon sequences were present at each coding region. Unlike the natural genetic code, each coding region used a set of codon sequences that were distinct from the codon sequences at the adjacent coding regions. Consequently, a total of 1536 different codon sequences were present in the library. The different codons at each coding position directed the addition of one amino acid from a set of seventeen different Fmoc-protected amino acids. An arginine dimer was included as an 18th amino acid in the fourth and final synthetic step, so some of the products were pentapeptides. An extra bar code (VE, black/white bar) specified whether the gene product would be subjected to a kinase substrate selection or to a control selection. Each peptide was coupled through a 5' polyethylene glycol linker to the gene that programmed its synthesis.

**Fig 3:**
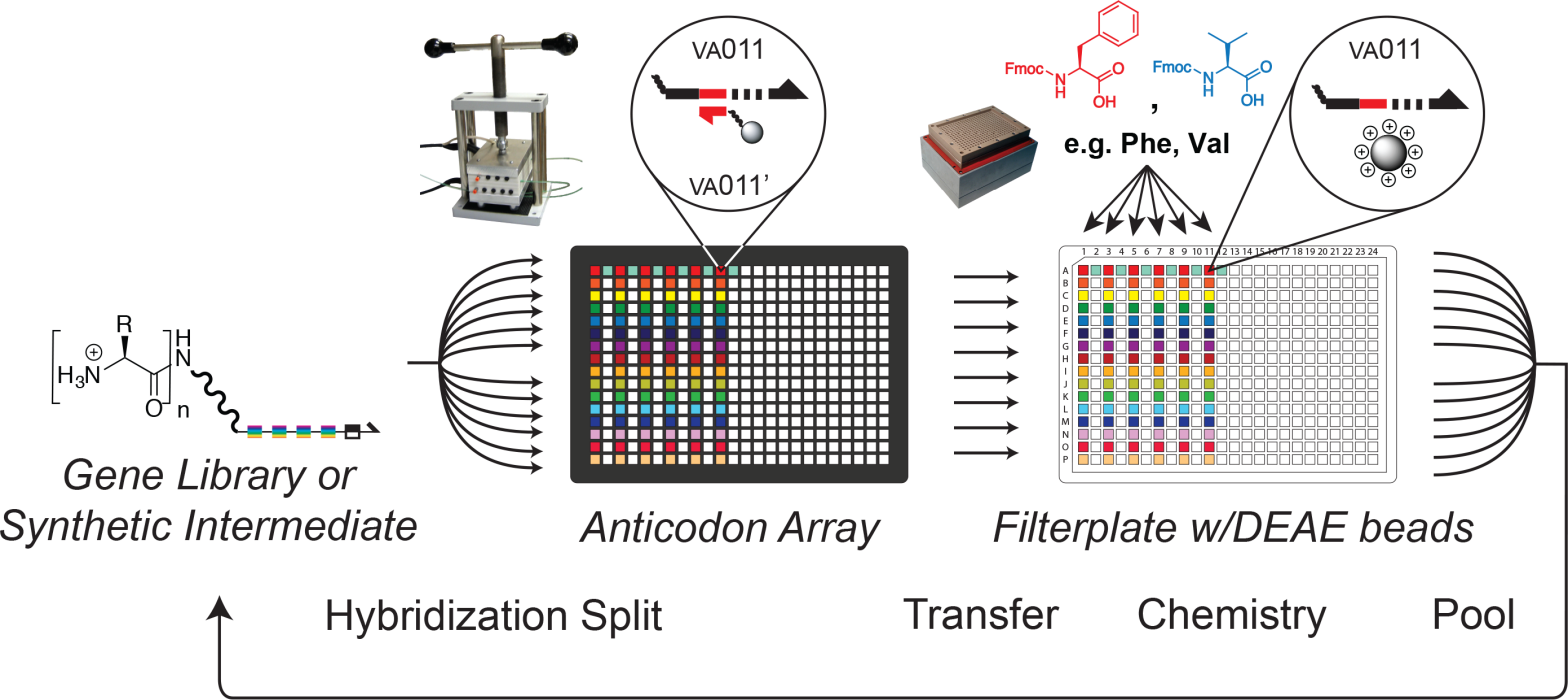
DNA-programmed combinatorial library synthesis. For each of four synthetic steps, the DNA genes were split into 384 sub-pools by hybridization of the codons in one of the coding regions to a spatially arrayed set of complementary oligonucleotides. The DNA genes were then transferred in a one-to-one fashion from the hybridization array into a 384-well filter plate loaded with DEAE-Sepharose resin. The DEAE resin acted as a solid support that retained the DNA genes during chemical reactions. One of seventeen different Fmoc-protected amino acids (dependent on the sub-pool position within the 384-well plate) was then coupled to the growing peptide chain linked to the DNA. After the chemical step, the genes were pooled, and the split-pool process was repeated until all of the coding regions had been chemically translated.

To close the evolutionary cycle, DNA must program small-molecule structures (Fig 1), which requires some form of DNA-programmed chemical synthesis. This has been accomplished by several different groups [19–21]. The transfer of traits from a parental small-molecule population to a child population has also been demonstrated, but only with small genetic codes (e.g. 10 building blocks, see references [19,21]). Directed evolution of small-molecule populations comprised of hundreds-to-thousands of building blocks remains unexplored. Such a large building-block alphabet is essential for low-molecular-weight chemical families, wherein compounds are formed from a highly diverse set of chemical fragments. With this in mind, we previously developed a DNA-programmed version of the traditional split-pool combinatorial library synthesis (Figs 2 and 3 and refs. [19,22]). We use this "chemical translation" process in a three-step directed chemical evolution cycle (Fig 1). The cycle corresponds closely to the practice of directed protein evolution. DNA routing and organic chemistry play the role of the ribosome, and synthetic small molecules are the evolving species. Recently, we reported mesofluidic devices for DNA-programmed combinatorial chemistry in a microplate format [23,24]. Using these devices, we have carried out a proof-of-concept experiment in which we program a small peptide library with a 384 letter code, and evolve it to obtain substrates of protein kinase A (Fig 1). We demonstrate the inheritance and amplification of fitness traits from an initial small-molecule population through to the great-grandchildren of that population. The results generalize the process of directed evolution to large genetic codes.

## Results

### Design of the Chemical Evolution Experiment

DNA-programming of an n-step chemical synthesis in microplate format can accommodate a library complexity of 384^n^. To explore the effectiveness of the method, we used this coding capacity redundantly. We designed a peptide library that was chemically assembled in four coupling steps, incorporating seventeen different amino acids in the first three steps, and eighteen in the fourth step (Fig 2). The eighteenth building block in the final coupling step was an arginine dipeptide, so that a portion of the peptides consisted of pentamers. Six DNA codons were assigned to each building block. Thus 2.17*10^10^ different DNA genes programmed the synthesis of a peptide, but only 88,434 unique tetramer and pentamer amino-acid sequences were produced because of redundancy in the genetic code.

The library was subjected to a selection for protein kinase A (PKA) substrates based on a previously described phosphopeptide-enrichment scheme (Fig 1B and ref. [25,26]). The DNA-peptide conjugate library was first incubated with protein kinase A and ATP gamma-S, and then treated with biotin iodoacetamide to alkylate thiophosphate moieties. Biotinylated DNA-peptide conjugates were isolated on streptavidin paramagnetic beads. Pilot experiments demonstrated 1000-fold enrichment of substrate-DNA conjugates over non-substrate conjugates (S1 Fig).

For the experiments described below, an internal-control selection was performed in parallel with the kinase selection. The control selection was designed to reveal gene enrichment caused by factors unrelated to the substrate proficiency of the encoded peptide substrates. It was identical to the substrate selection except that the kinase protein was replaced with buffer. The control DNA-peptide conjugates were distinguished from the kinase-treated DNA-peptide conjugates by a barcode inserted into the DNA genes (Fig 2). The two pools of genes were combined and chemically translated together. They were then separated from each other after library synthesis by hybridization of the bar-code sequences to two different oligonucleotide affinity resins.

### Large-scale Identification of PKA Substrates

The library of DNA-peptide conjugates was evolved over four generations. Each round consisted of a chemical translation step, the application of selective pressure favoring kinase substrates, and then the amplification and diversification of the enriched genes (Fig 1). The gene populations of the second, third and fourth generations were sequenced and analyzed. Gene enrichment was calculated as the ratio of a gene's fractional abundance in the kinase-treated population relative to its apparent fractional abundance in the mock-treated control population.

**Fig 4:**
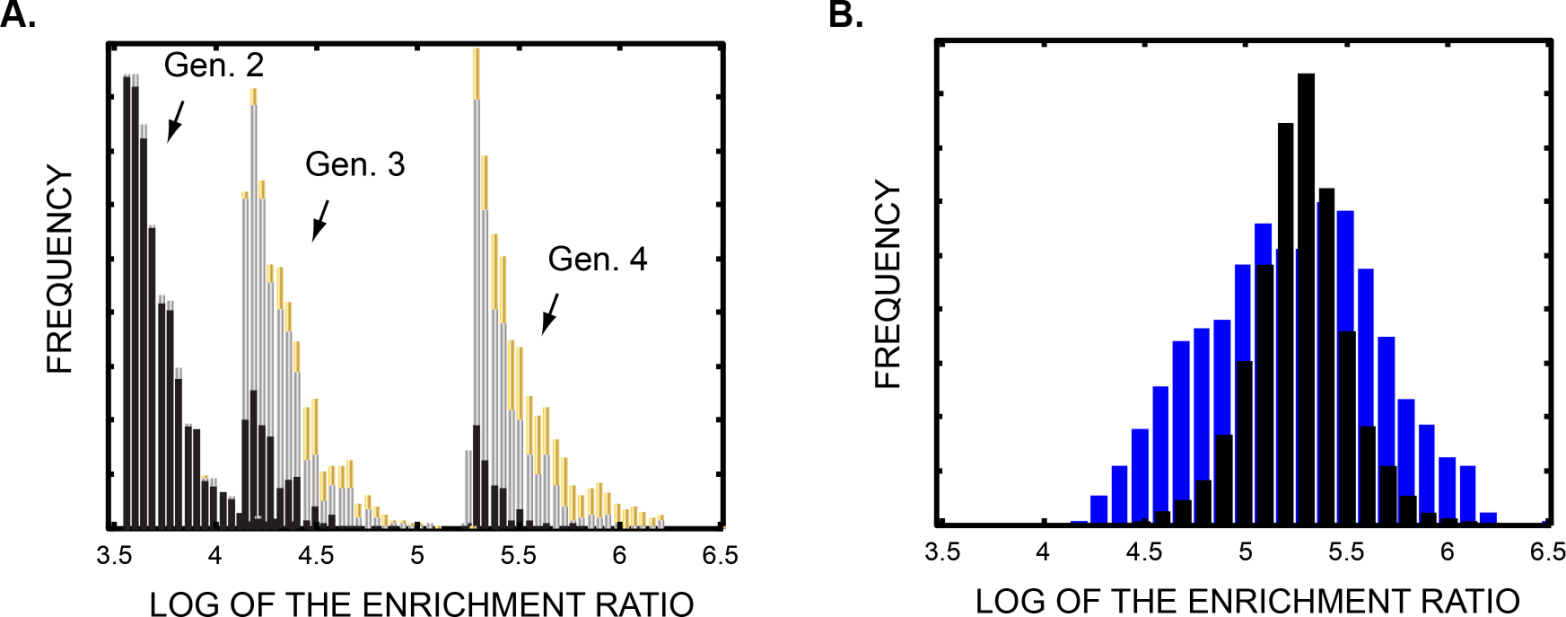
Population maturation. **A.** The peptide-DNA conjugate library converged to PKA substrates over four generations. A histogram of the fold-enrichment ratios for the top 1000 genes in generations 2–4 is shown. Genes lacking a consensus motif are colored black, genes that encoded peptides with one of the two PKA consensus motifs (RR*[S/T]* or RRSF*) are colored silver, and genes that encoded the top RRSFL peptide are colored gold. See also S4 Fig. **B**. The DNA sequence of genes with synonymous codon substitutions influenced the enrichment of peptide-DNA conjugates. A histogram plot with blue bars shows the observed distribution of log fold-enrichment ratios for 830 different genes that encoded the same RRFSL peptide (95% are contained between 4.4 and 6). If all of the RRSFL-encoding genes had been equally enriched, the black distribution would have been expected (this computed distribution reflects Poisson noise from sparse gene sampling). The excess width of the observed distribution suggests the existence of a selection bias for or against different synonymous codons. The Poisson distribution was reduced to 0.63 of its full area for clarity of the plot.

By the fourth generation, 999 of the 1000 most abundant genes encoded a serine or threonine residue as well as an N-terminal arginine (versus 3 expected by chance; see Fig 4A). Between the second and third generations, many individual substrate-encoding genes had become sufficiently enriched to appear at least twice in ~3 million sequencing reads. That threshold required a 15,000-fold cumulative enrichment, corresponding to a 10–50 fold enrichment of substrate-encoding genes per selection step.

To identify the most highly enriched peptides, we summed the reads for the 6^4^ (1296) different genes corresponding to each peptide sequence. The dominant sequence motifs in the fourth generation data set were RRX[S/T]* and RRSFX, where the letter X is generally a hydrophobic residue (see Table 1 and S2 Fig). The first motif corresponds to an established consensus sequence for protein kinase A [27–30]. The second motif is unusual among known substrates [30]. We measured k_cat_/K_M_ values for nine peptides with diverse enrichment rankings to compare the sequencing data with substrate proficiencies (Table 1). Peptides in the alternate class were phosphorylated by PKA, but their phosphorylation was less efficient than for peptides in the canonical class. Notably, two of the evolved pentapeptide mini-substrates were phosphorylated more rapidly than the Kemptide heptapeptide, which is considered an optimal PKA substrate [27]. One noncanonical tetrapeptide weighing 563 daltons had a k_cat_/K_M_ value only 28-fold lower than that of Kemptide.

**Table 1:** k_cat_/K_M_ values for resynthesized peptides.

Peptide^[^^a^^]^	Rank^[^^b^^]^	LTFE^[^^c^^]^	Rel. k_cat_/K_m_^[^^d^^]^
RRSFL	1	5.4	0.012
RRSFV	7	4.8	0.013
RRASL	20	4.6	1.1
RRFSV	33	4.4	4.1
RRMSV	51	4.1	2.2
RRMTV	95	3.7	0.031
RMSF	147	3.3	0.0028
RRSF	253	2.7	0.035
RRMS	1846	1.3	0.028
LRRASLG^[^^e^^]^	---	---	1

The peptide sequences are written from the N-terminus to the C-terminus in the single-letter amino-acid code (A-Alanine, F-phenylalanine, L-leucine, M-methionine, R-Arginine, S-serine, T-threonine, V-valine). See also S2 Fig for consensus sequences of evolved substrates.

^a^Peptides were synthesized as C-terminal amides.

^b^Rank: Position on list of peptides ranked by fold enrichment, calculated by summing reads over the 1296 genes encoding each peptide.

^c^LTFE: log (total fold enrichment) after four rounds.

^d^k_cat_/K_m_ measurements are relative to Kemptide. The SEM values are ~30% of the reported mean value based on three independent measurements.

^e^Kemptide peptide was a C-terminal carboxylate.

### Population Behavior Under Selective Pressure

To estimate the discovery power of our directed evolution system, we analyzed the data as though each codon represented a distinct amino acid. This models a fully complex chemical library, in which 384 different chemical building blocks are used in the library synthesis. Accordingly, each gene was treated as though it produced a unique peptide gene product, rather than as one of 1296 genes that encoded the same amino-acid sequence. We first examined the enrichment of the 1296 individual genes that encoded RRSFL, the top-ranked peptide hit. Ideally, the enrichment of all 1296 genes would have been identical because they all encode the same peptide product. As illustrated in Fig 4B, the observed enrichments varied from 28,000-fold to one million-fold with a median enrichment of 175,000-fold (Fig 4B). Poisson noise accounted for one third of the variance in the distribution. The additional variance indicates that the DNA sequence of the genes influenced enrichment. These data reflect a selection bias for or against specific DNA codons that is independent of the identity of the encoded amino acid. During the transmission of genes over a single generation, the two-sigma variation in log-enrichment due to codon bias was ~1/9 of the median log-enrichment.

**Fig 5:**
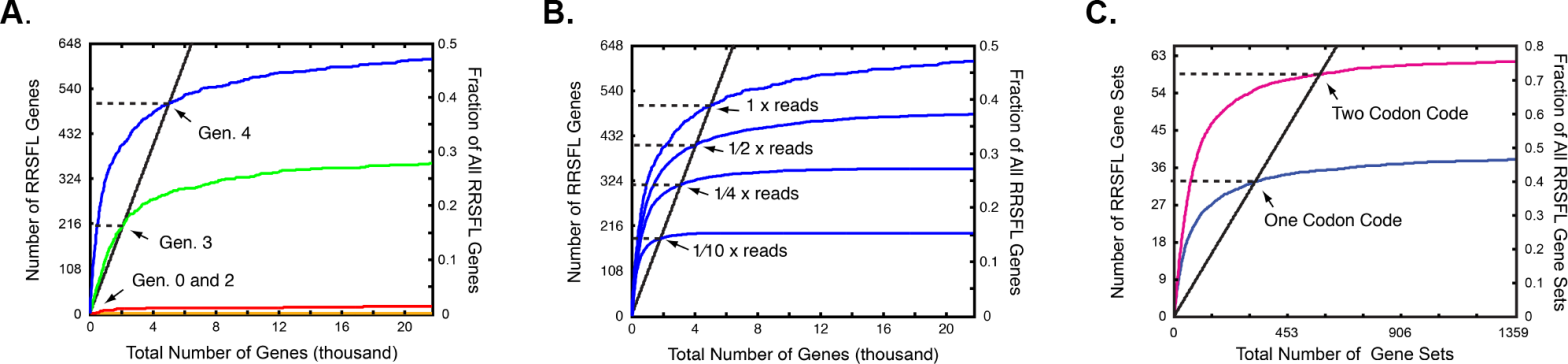
Accuracy of hit detection. The plots show the number of RRSFL encoding genes (y-axis) contained within the top N total genes (x-axis) of a list ranked by enrichment ratio. If the gene ranking had been perfect, the curves would have gone straight up the y-axis and then cut right on the x-axis at the top of the plot. The solid black line shows how RRSFL genes accumulate at a 90% false discovery rate (i.e. when every tenth gene is a hit). The y-value at the intersection of each curve with the solid black line corresponds to the number of RRSFL genes that would have been detected below a 90% false discovery threshold. **A.** Improved gene ranking over successive generations. The number of RRSFL genes at the top of ranked lists from the zeroth (yellow), second (red), third (green) and fourth (blue) generations is shown. None of the RRSFL genes could be detected below a 90% false discovery threshold in the zeroth or second generations, whereas 207 and 505 out of 1296 total could be detected in the third and fourth generations respectively. **B.** Dependence of gene ranking on sequencing depth. The effect of using increasingly small fractions of the total sequencing data to rank genes is shown. 505, 416, 319 and 188 of the RRSFL genes could be detected below a 90% false discovery threshold given 3 million, 1.5 million, 0.75 million and 0.3 million sequencing reads respectively. The discovered fraction of RRSFL genes grew roughly in proportion to the square root of the number of reads. **C.** Improved gene ranking with a redundant genetic code. The ranking of RRSFL gene sets based on 187500 gene reads and a two codon-per- amino acid genetic code is shown. In one case, the reads used for the analysis were restricted to genes containing a single codon from each codon pair. In this single-codon case, 32 of the 81 RRSFL genes sets could be detected below a 90% false discovery threshold. Alternatively, an identical number of gene reads were used for the analysis, but the reads included genes containing both codons of each codon pair. In the two-codon case, 58 of the 81 RRSFL genes sets could be detected. The two-codon genetic code revealed 70% of the RRSFL gene sets, while the one-codon code revealed only 40%.

We next examined how codon bias influenced the ranking of RRSFL-encoding genes relative to all other genes. Importantly, accurate ranking impacts the utility and efficiency of directed chemical evolution because the synthesis and screening of individual candidate molecules is the time and resource-limiting step. Ideally, the RRSFL-encoding genes should have been stacked at the very top of the rank order list. In reality, only half of them were present in the top 20 thousand genes, which is the top 1 part per million of all genes (Fig 5A). If one had made and tested the encoded peptides descending from the top of the ranked gene list, 505 of the 1296 RRSFL-encoding genes would have been discovered before hitting a 90% false discovery rate.

A key question is how the accuracy of small-molecule ranking depends on the design parameters of the evolutionary system: specifically the number of generations over which the population is evolved, the sequencing depth, and the nature of the genetic code (Fig 5). Our experimental data provide an opportunity to address these questions. Again, as a metric of ranking accuracy, we used the number of RRSFL-encoding genes that appeared above a 90% false discovery threshold. Fig 5A shows the location of hit genes in a ranked list of 20 thousand genes from the second, third and fourth generation libraries. With the 90% false discovery cutoff, 0, 207 and 505 hit genes were found in the second through fourth generations. Thus, the accuracy of hit detection improved dramatically in child generations relative to their parents. Most of the top ranked genes in the second generation did not encode a kinase substrate: over 60% of the encoded peptides lacked a serine or threonine residue, and an additional 30% lacked the ubiquitous arginine residue recognized by protein kinase A. The emergence of substrates out of the noise over successive generations resembles the population behavior observed in directed protein evolution.

We estimated the effect of read depth on ranking accuracy by comparing rank predictions from increasingly small subsets of the sequencing data (3*10^6 reads down to 3*10^5 reads, Fig 5B). The fraction of hits discovered below the 90% false discovery threshold decreased from 0.43 to 0.15 with a ten-fold reduction in the number of reads. More generally, the fraction of the hits that were discovered was proportional to the square root of the number of reads. This relationship is expected if Poisson noise in the population sampling obscures differences in gene fitness.

In principle, genetic code redundancy should reduce the spurious influence of gene DNA sequence on peptide enrichment. To explore this, we modeled a hypothetical case in which two codons programmed each chemical building block. We broke the six codons specifying each amino acid into three pairs of two codons. Thus, the 1296 genes encoding each peptide were assigned to 81 sets containing 16 (2^4) genes each. We compared two cases. In the first case, we eliminated one codon of each codon pair and threw out all of the sequencing reads that contained the eliminated codon. Thus, only one codon of each pair remained in the data set to represent the associated amino acid. Alternatively, we kept both codons in each pair, but threw out 15/16th of the sequencing reads. Given exactly the same number of reads (roughly 187500), only 32 of the 81 RRSFL-encoding gene sets could be recovered with one codon per amino acid, whereas 58 of the 81 gene sets could be recovered with two codons per amino acid (Fig 5C). The two-fold redundant genetic code markedly improved detection of the hit gene sets.

## Discussion

Ultimately, the purpose of directed evolution is to identify functional molecules hidden within large chemical spaces. Detection sensitivity is a function of the complexity of the chemical space, the depth of the sequencing reads, and the fold enrichment conferred by the selection. These factors are linked by the fact that a single gene must appear at least twice in a sequencing data set to be distinguished from noise. Our library contained 20 billion genes, and we collected three million reads per sequencing run. Thus single genes had to be enriched 15000-fold on average to generate an interpretable enrichment signal, i.e. to give an expectation value of two reads. Sensitivity could be improved by increasing the read depth, where coverage appeared to improve as the square root of the number of reads. Alternatively, sensitivity was improved by exponential amplification of fold-enrichment ratios over multiple generations.

It has been observed experimentally that increasing the diversity of the building blocks that comprise a chemical space improves the quality of the hits that are discovered [31–36]. A great variety of building blocks is needed to achieve high diversity in small-molecule libraries because they are subject to molecular-weight restrictions. Large chemical alphabets and the genetic codes that program them are therefore necessary. Directed evolution with alphabets consisting of hundreds of building blocks is currently uncharted territory for either biological or synthetic molecules. The data reported here provide a first glimpse of molecular evolution in this new regime. The approach we report should be applicable to a range of chemical families, as libraries of macrocycles, peptoids, triazines, bicyclosuccinimides, benzimidazoles and pyrimidines have already been synthesized in a DNA-linked format [14,15,20,22,37,38]. Many standard synthetic transformations have been adapted for DNA-linked chemistry (see ref. [39]), which will provide access to new structural classes.

As with biopolymer evolution (see ref. [40]), it is possible for gene sequences to spuriously influence the results of a directed chemical evolution campaign. For example, we observed that different genes encoding the same peptide sequence were not uniformly amplified. This is probably related to the fact that the fractional abundance of the 1536 codons in the control gene population changed significantly over four generations (S3 Fig). The changes in codon abundance must also have caused enrichment and depletion of genes in the kinase-selected population, which may not have been adequately corrected for. The skewing of codon abundances was not due to depletion by chemical modification of the DNA, because codons directing an amino-acid coupling step were enriched relative to codons directing a blank step (S3 Fig). Amplification bias also seems unlikely based on the gene design. Each 20mer coding region was flanked by common 20mer constant regions, so that all of the genes were at least 55% identical and used identical PCR primers. The changes in codon abundance were most likely caused by differences in hybridization efficiency. Ultimately, a minimal level of redundancy in the genetic code effectively mitigated the effects of gene-sequence artifacts by averaging out changes in codon abundance.

Variations in synthetic yield complicate the interpretation of enrichment ratios, because synthetic yields prorate the true enrichment ratio. Unlike polymerase and ribosome-mediated biosynthetic processes, chemical synthesis almost never proceeds with 100% yield. This is an important distinction between the directed evolution of proteins and the directed evolution of small molecules. All else being equal, small molecules made in high yield will trump those made in low yield. Importantly, it should be possible to disentangle functional fitness and synthetic yield by varying the selection stringency. High yielding but less functional molecules should exhibit the largest enrichment ratios at low stringency, but their enrichments should decrease as selection stringency is increased. Conversely, low yielding but highly functional molecules should not exhibit the highest enrichment ratios at low stringency, but their enrichments should improve as stringency is increased. Aside from pro-rating enrichment, poor synthetic yields can also complicate the analysis of hits, which may require deconvolution of a heterogeneous product mixture.

The emergence of PKA substrate motifs in our experiment (Fig 4A) demonstrates that kinase substrate proficiency was the dominant driving force for enrichment. However, k_cat_/K_M_ did not correlate tightly with enrichment ranking, suggesting that the selection process was not fully aligned with the desired molecular property. Differences between peptides in the rate of thiophosphate alkylation (the second step of the selection procedure) may have unintentionally contributed to apparent fitness, favoring faster-alkylating sequences. We speculate that interactions between the thiophosphorylated serine residue and the neighboring cationic amino acids influenced alkylation rates, as has been observed for the alkylation of cysteine residues [41,42]. Additionally, the stringency of the enzymatic reaction step was too lax. Based on literature kinetic constants [25,43] and the conditions of the enzyme treatment, the half-life for kemptide thiophosphorylation should have been roughly one minute. During the one-hour protein kinase A incubation, substrates with catalytic proficiencies much worse than kemptide would have been significantly phosphorylated. Insensitivity to functional fitness in the desired range opened the door for other characteristics to impact enrichment.

Our experiments illustrate several roles for an evolutionary cycle that acts on a DNA-tagged combinatorial library. First, the cycle provided exponential amplification of hits despite a selection process with a low ceiling on fold enrichment. The maximum single-generation enrichment ratio we observed was 50-fold, but thousands of genes were enriched by more than 10^5^ fold in the fourth generation. The cumulative enrichment provided a fitness landscape with hundreds of peptides, rather than a sparse list of hits. It is likely that small enrichment ratios will be a universal problem with DNA-tagged combinatorial libraries, because low product yields from challenging synthetic chemistry are unavoidable and will prorate true enrichment. The evolutionary cycle also made it possible to iteratively run a "single-shot" selection strategy, meaning a selection strategy that cannot be repeated without library resynthesis. In our case, testing for kinase substrates introduced two irreversible covalent modifications, thiophosphorylation and alkylation with biotin iodoacetamide. Repeating the selection required a pristine population of molecules that had not been chemically modified in a previous selection step. This would not have been possible without DNA-programmed chemistry. There are many other types of single-shot selections that are highly valuable, for example selection for ligands to receptors that are overexpressed in intact cells. Finally, the evolutionary cycle made it possible to generate exceptionally fit hybrids by recombination of enriched genes between generations. This enabled the "genetic algorithm" for functional optimization, a process that has proved extremely powerful in the context of directed protein evolution (see e.g. ref. [44]). Although library diversification between generations was not necessary in our experiment, because the limited library diversity was oversampled, it will be essential for complex chemical spaces that cannot be adequately sampled in a single generation.

Natural products are the fruits of bio-combinatorial chemistry and chemical evolution that occurred over billions of years. Enzymes carried out the small-molecule synthesis and a diverse array of metabolites served as building blocks. Here we have demonstrated a gene translation system that accommodates extremely large genetic codes, making it possible to reenact the chemical-evolution process in a test tube. With unlimited synthetic possibilities and universal building-block alphabets, evolution is sure to yield surprises. Hopefully, these will include molecules with remarkable functional properties, akin to the natural products which inspired this work.

## Materials and Methods

### Reagents

Solvents and general chemistry reagents were purchased from VWR International (West Chester, PA), Fisher Scientific (Hampton, NH), or Sigma-Aldrich (St. Louis, MO). Fmoc protected amino acids were from Novabiochem (La Jolla, CA) or Chem-Impex (Wood Dale, IL). Oligonucleotides were purchased from Bioneer (Alameda, CA) and the Stanford PAN Facility (Stanford, CA). The catalytic subunit of murine cAMP-activated protein kinase A was from NEB (P6000L) and peptides were obtained from AnaSpec (Fremont, CA) or Thermo Scientific Pierce Protein Research (Rockford, IL).

### DNA-programmed Combinatorial Chemistry

DNA-programmed combinatorial chemistry derives from the split-pool technique for synthesis of combinatorial small-molecule libraries on polystyrene beads. There are a few key differences. First, a short DNA fragment (the "gene") replaces each polystyrene bead, and the DNA fragment acts as the solid support. A molecule is synthesized at the end of a linker attached to the DNA fragment. For the experiments reported here, there is only a single copy of the small molecule associated with each fragment. Chemical reaction steps are performed while the DNA fragment is absorbed onto anion-exchange beads, so that conventional filter-based strategies for solid-phase synthesis can be used. Second, the splitting step is fundamentally different. Whereas the polystyrene bead supports are split randomly into different reaction vessels in a conventional split-pool approach, the DNA-fragment supports are split deterministically into different reaction vessels in the DNA-programmed approach. Specifically, each DNA fragment is routed to a single position of a 384-microcolumn array (the "anticodon array") because a short sequence within the DNA fragment hybridizes to a complementary oligonucleotide immobilized on one microcolumn in the array. The routed DNA fragments are then transferred from the microcolumns into the corresponding wells of a 384-well filterplate. The filterplate contains anion-exchange resin beads that bind to and retain the DNA fragments. The wells of the filterplate then serve as independent chemical reaction vessels for coupling reactions. A different synthon is used as the reactant in each well. Thus, because the nucleotide sequence of each DNA fragment determines the reaction vessel it winds up in, the nucleotide sequence also determines the chemical transformation to which the fragment will be subjected. The process of routing and chemical coupling is repeated for each step in a multi-step synthetic pathway. For a four-step synthesis, for example, four distinct sub-sequences within each DNA fragment determine the four microplate positions that the fragment will adopt during the library synthesis. By determining these microplate positions, each DNA fragment programs a sequence of four chemical transformations. This synthetic sequence produces the small molecule to which the fragment is ultimately attached. Operationally, the DNA fragment plays the same role that an mRNA fragment plays in the process of protein synthesis.

The amino-acid coupling steps used here were carried out using EDC and HOAt in methanol or in DMF. Although these solvent conditions are incompatible with DNA solubility, the reactions proceed efficiently while the DNA is absorbed onto anion exchange beads. Coupling yields have been measured previously to be 85–100% [45].

The workflow for programmed library synthesis, comprising a series of DNA-directed library splitting steps followed by chemical coupling steps, was carried out as previously described [19,22]. For the splitting steps, the ssDNA library was hybridized to an anticodon array overnight at 37°C using a mesofluidic hybridization pump [23]. The anticodon array with bound DNA was then mounted into a 384-well adapter device [23] which uses rubber gaskets to form an isolated liquid channel above and below each array feature. The adapter device was placed on top of a 384-well polypropylene filter plate with 5 μm glass fiber frits (E&K Scientific, EK-2287) containing 5 μl aliquots of DEAE sepharose resin. DNA was eluted from each feature of the anticodon array onto the DEAE resin in the corresponding well of the filter plate by application of a denaturing buffer (10 mM NaOH with 1 mM EDTA and 0.005% Triton X-100) followed by gentle centrifugation of the stack (140xg for 1 minute). The DEAE resin in the filter plate was then washed three times with 85 μl H_2_O and three times with 85 μl dry methanol. Peptide couplings were performed as previously described using EDC and HOAt in methanol and DMF [45]. Following the synthetic steps, the filter plate was placed on top of a 384-well polypropylene microtiter plate, and DNA was eluted from the DEAE support by application of a high salt buffer (1.5 M NaCl, 50 mM NaOH, 1 mM EDTA, and 0.005% Triton X-100) followed by gentle centrifugation. The eluted DNA was pooled, concentrated and buffer exchanged into hybridization buffer using a centrifugal filter device with a 10,000 Da molecular weight cut-off (GE Healthcare). BSA and tRNA were added to 0.5 mg/ml each. The sample was diluted to 3 ml with hybridization buffer and applied to another anticodon array. Following the final step of the chemical synthesis, the eluted DNA was split by hybridization for 3 hours at 37°C to a pair of anticodon columns derivatized with the VE_001_ and VE_002_ anticodon sequences. The two halves of the library were concentrated with n-butanol extractions, precipitated with isopropanol, and subjected to a kinase-substrate selection or a control selection, respectively.

### Selection for Protein Kinase A Substrates

Translated libraries were incubated in PKA buffer (NEB) overnight at 30°C with 10,000 units of protein kinase A and 1 mM ATPγS. For the selection between the third and fourth generations, the incubation was shortened to 1 hour at 30°C. Control selections were performed identically, with the enzyme replaced by a 50% glycerol solution. After incubation, the crude reactions were diluted 1.5-fold and adjusted to 100 mM NaOAc pH 5.2 and 25% dimethyl formamide. EZ-Link iodoacetyl-LC-biotin (Pierce Thermo Fisher Scientific, 21333) was added to 600 μM. After 3 hours in the dark at 25°C, the treated library was cleaned up by extraction with phenol:chloroform and n-butanol, and then precipitated with isopropanol. The library was resuspended in 27.5 μl bind buffer (10 mM Tris pH 7.4, 1 mM EDTA, 1 M NaCl), and 1.25 mg/ml tRNA and 0.25 mg/ml BSA were added. The library was then incubated with 15 μl of μMACS streptavidin microbeads (Miltenyi Biotec, 130-074-101) overnight at room temperature. The microbeads were purified and washed over MACS columns according to the manufacturer’s instructions. The beads were eluted, and the purification process was repeated a second time on a fresh MACS column. DNA was amplified directly off of the streptavidin beads by PCR. Between generations, the library DNA was diversified by recombination, so that 2–5% of the genes in the child library were chimeras of genes selected from the parental library.

### Illumina Sequencing

25 pmol of amplified DNA was used as a template in a PCR reaction that appended Illumina adaptor sequences. The PCR product was quantified on an Agilent 2100 Bioanalyzer, and a 10 nM solution was submitted to the Stanford Functional Genomics Facility for sequencing using custom primers. Paired-end 150-bp reads were obtained on a MiSeq Illumina Sequencer.

### Data Analysis

The sequencing data were processed using scripts written in either AWK or MATLAB. First, a string search on the fastq MiSeq files was used to locate constant-region sequences (Z_a_-Z_f_). The 20 base-pair blocks adjacent to each constant region were excised and saved. The forward reads gave sequences for the first four codons (A-D), and the reverse reads gave reverse-complement sequences for the last five codons (B-E). Redundant forward and reverse reads were obtained for the three central codons (B-D). The 20mer blocks were converted into codon numbers using a direct string comparison to each of the 384 possible codon sequences. In order for a codon number assignment to be made, at least 18 bases had to match between the observed 20mer and the reference codon sequence. Paired reads that any gave contradictory assignment at codons B-D were discarded. The raw list of codon sequences was sorted, and the reads were summed, to generate a non-redundant list of codon sequences and the number of times that each sequence was observed in the data. The codon-sequence data were then split into a kinase-selected block and a mock-selected block based on the identity of the E codon. The codon frequencies at each coding position in the mock-selected library were calculated, and the fractional abundance of each four-number codon sequence in the mock library was approximated as the product of the frequencies of its constituent codons. The fold-enrichment ratios of genes with two or more reads in the kinase-selected library were calculated as the ratio of the gene's fractional abundance in the kinase-treated population relative to its apparent fractional abundance in the mock-treated control population. For the analysis with a reduced number of reads, a subset of the total reads was selected randomly. The calculations were repeated 50 times with different random subsets, and the 50 receiver-operator characteristic curves were averaged. For the analysis based on a two codon per amino acid genetic code, synonymous codons were paired randomly. The calculations were repeated 50 times with different random pairings, and the 50 receiver-operator characteristic curves were averaged. The distribution of log fold-enrichment ratios expected by chance from a finite DNA sequencing sample was computed based on 3 million gene reads, an assumed enrichment of 175,000 for all RRSFL-encoding genes, and the prior codon abundances observed in the control gene population.

### K_cat_/K_m_ Measurements

Candidate peptides were synthesized as C-terminal amides. Phosphorylation of the peptides by protein kinase A was measured using a P81 phosphocellulose filter binding assay with ^32^P-ATP [46]. Substrate concentrations were normalized to the kemptide concentration by coupling each peptide to fluorescamine, separating the peptide from unreacted fluorescamine on a high-pressure liquid chromatography column, and integrating the signal from a fluorescence detector. Substrate concentrations were additionally verified by measuring the radioactive signal following exhaustive peptide phosphorylation (overnight incubation with 40 nanomolar PKA). Initial phosphorylation rates were measured at peptide concentrations between 0.1 and 5 micromolar, a protein kinase concentration of 0.4 nanomolar, and ATP concentration of 10 micromolar for incubation times between 10 minutes and one hour. In all cases, the observed initial rates increased linearly with peptide concentration. The data were used to fit a second order rate constant relative to the kemptide standard.

## Supporting Information

S1 Fig (Related to Fig 1): Fold-enrichments for model peptide-DNA conjugates. Control peptide-DNA conjugates were spiked into an excess of background DNA, and then subjected to the biochemical selection that enriches for protein kinase A substrates. The fractional abundances before and after selection were measured by quantitative PCR.
(TIF)

S2 Fig (Related to Table 1): Consensus sequences of evolved substrates. Sequence logos for the two classes of peptides encoded by highly enriched genes selected for PKA substrates. 5-mer peptides with log-enrichment ratios greater than 2 were binned into the appropriate substrate class. Peptides were weighted by their enrichments and sequence logos were prepared using weblogo.berkeley.edu/logo.cgi. **Top**: The most highly enriched class of substrates with no intervening residue between the serine/threonine residue and the two arginine residues. **Bottom**: Substrate class matching the consensus sequence for PKA.
(TIF)

S3 Fig (Related to Fig 4): Concordance of codon abundance. Codon abundance in the initial DNA population is plotted against codon abundance in the fourth generation mock-selected population. Codons specifying an amino-acid coupling step are green. Codons specifying a blank (no chemistry) step are blue.
(TIF)

S4 Fig (Related to Fig 4): Concordance of single-gene enrichment rank with true fitness rank. Values are shown for the 10829 genes with ≥10 reads. True fitness rank is the rank of the encoded peptide. The peptide ranks were determined by summing reads over all of the 1296 genes that encoded each peptide. More than 98% of the single genes encode a peptide product with a true fitness rank below 100. Twenty extreme outliers with spurious single-gene enrichments are evident. The corresponding peptide products all include two or more blank building blocks.
(TIF)

S1 File Supporting Experimental Procedures with six supplemental references. (PDF)
