# Supplementary figures and images for: Directed Chemical Evolution with an Outsized Genetic Code
Source: PLoS One. 2016 Aug 10;11(8):e0154765. doi: 10.1371/journal.pone.0154765 (PMC4980042; doi:10.1371/journal.pone.0154765)

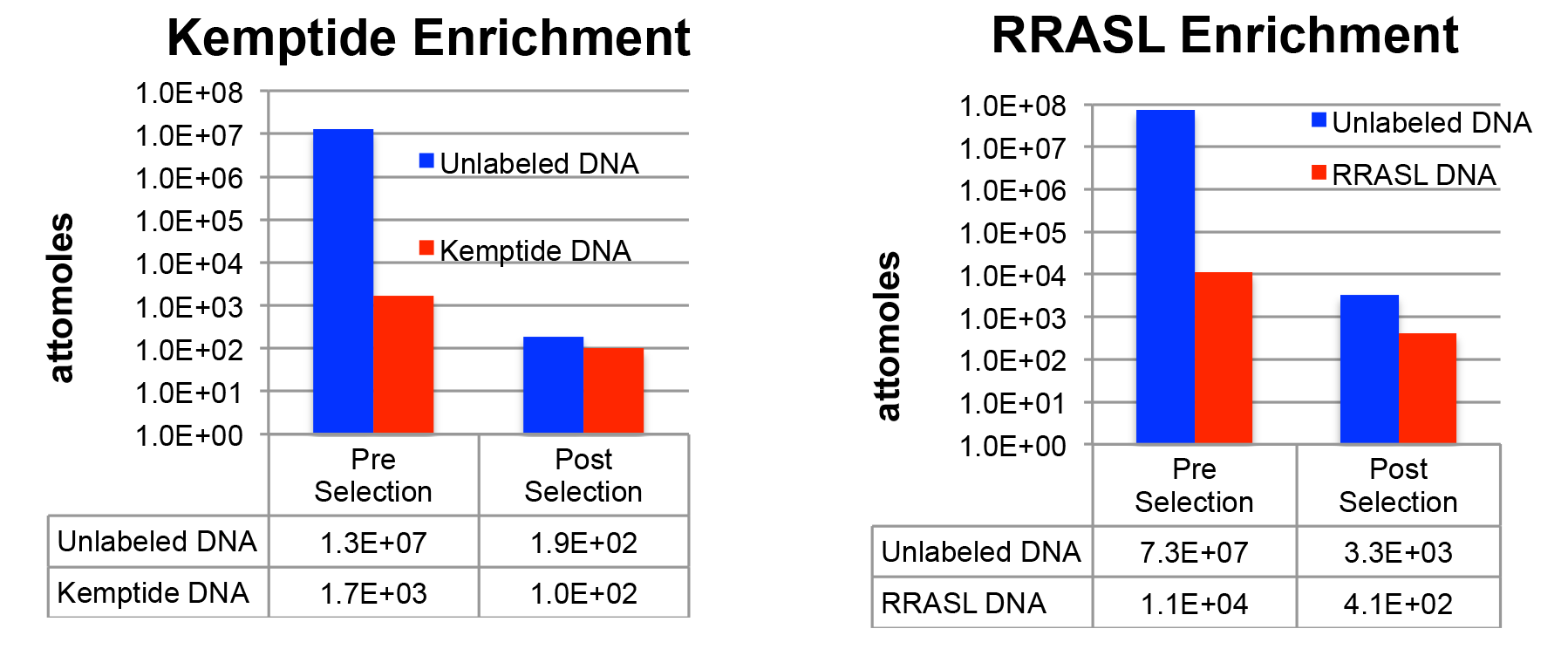

Supplement: S1 Fig — Control peptide-DNA conjugates were spiked into an excess of background DNA, and then subjected to the biochemical selection that enriches for protein kinase A substrates. The fractional abundances before and after selection were measured by quantitative PCR. (TIF) [file pone.0154765.s001.tif]

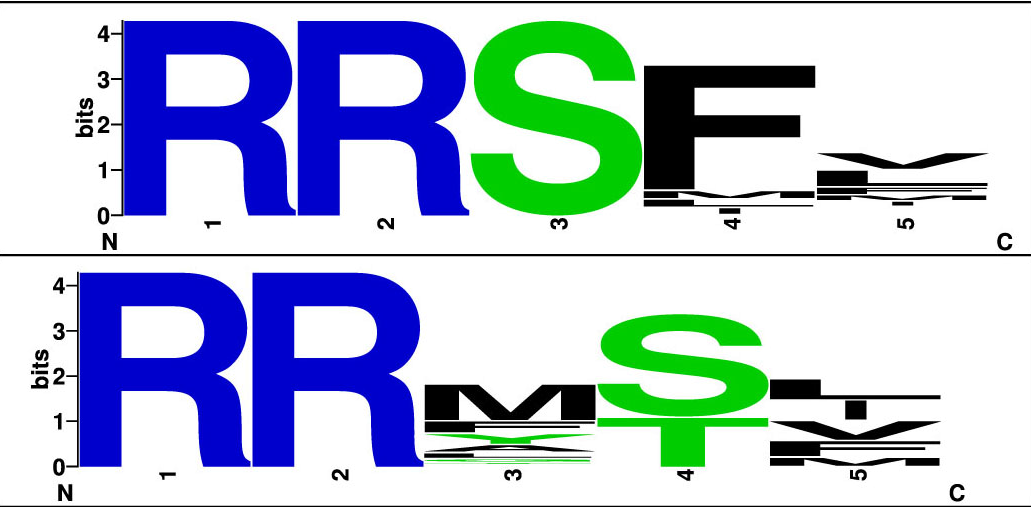

Supplement: S2 Fig — Sequence logos for the two classes of peptides encoded by highly enriched genes selected for PKA substrates. 5-mer peptides with log-enrichment ratios greater than 2 were binned into the appropriate substrate class. Peptides were weighted by their enrichments and sequence logos were prepared using weblogo.berkeley.edu/logo.cgi. Top: The most highly enriched class of substrates with no intervening residue between the serine/threonine residue and the two arginine residues. Bottom: Substrate class matching the consensus sequence for PKA. (TIF) [file pone.0154765.s002.tif]

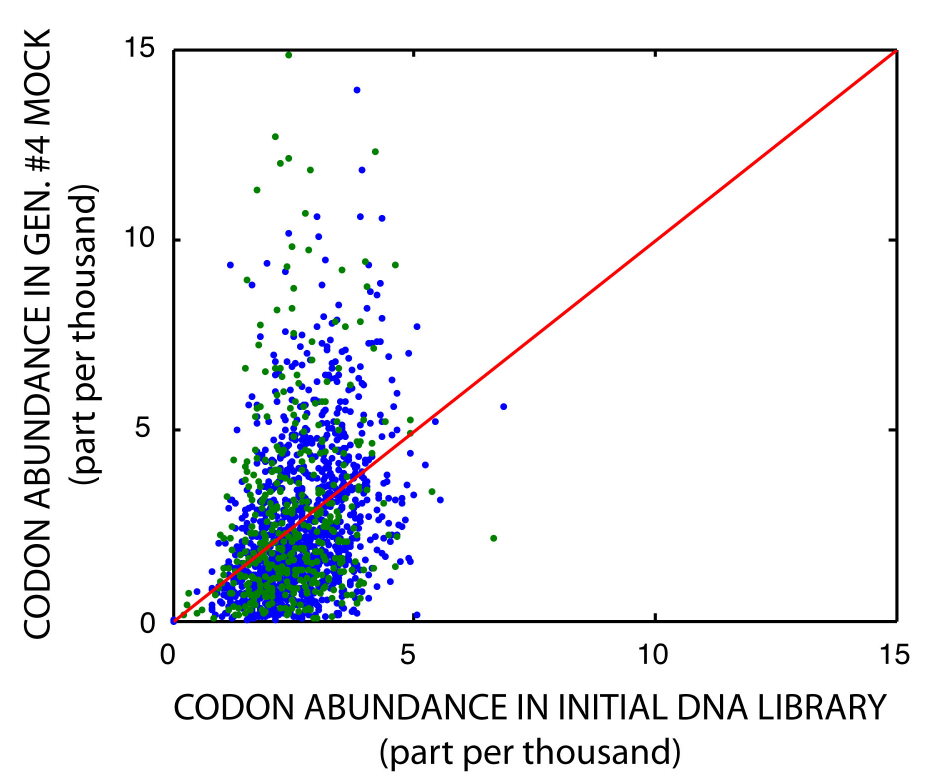

Supplement: S3 Fig — Codon abundance in the initial DNA population is plotted against codon abundance in the fourth generation mock-selected population. Codons specifying an amino-acid coupling step are green. Codons specifying a blank (no chemistry) step are blue. (TIF) [file pone.0154765.s003.tif]

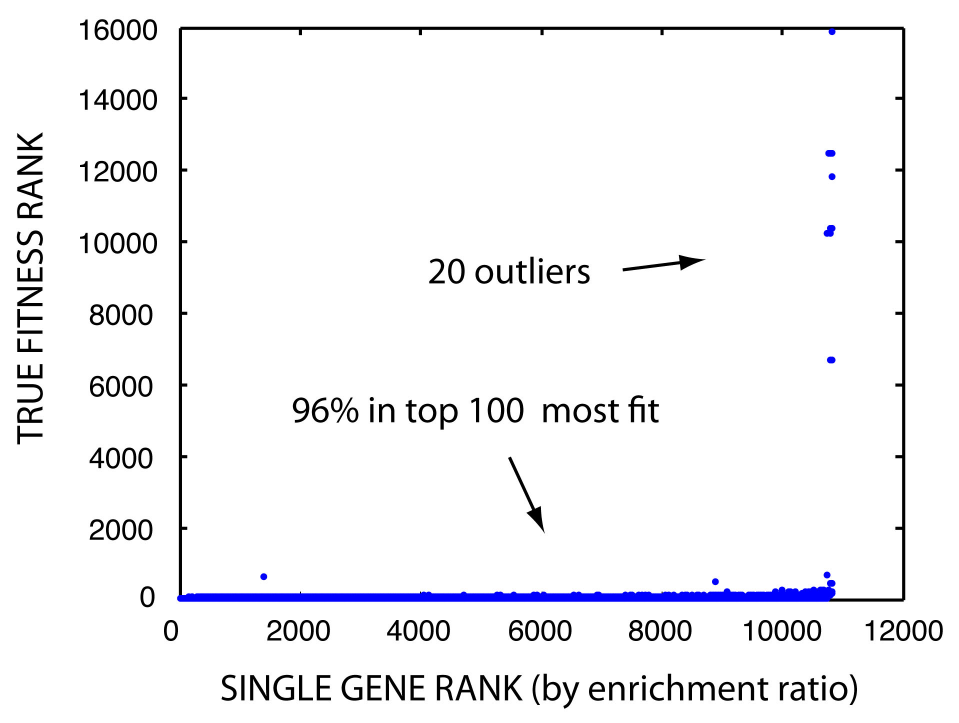

Supplement: S4 Fig — Values are shown for the 10829 genes with ≥10 reads. True fitness rank is the rank of the encoded peptide. The peptide ranks were determined by summing reads over all of the 1296 genes that encoded each peptide. More than 98% of the single genes encode a peptide product with a true fitness rank below 100. Twenty extreme outliers with spurious single-gene enrichments are evident. The corresponding peptide products all include two or more blank building blocks. (TIF) [file pone.0154765.s004.tif]
