# Supplementary material for: Directed Chemical Evolution with an Outsized Genetic Code
Source: PLoS One. 2016 Aug 10;11(8):e0154765. doi: 10.1371/journal.pone.0154765 (PMC4980042; doi:10.1371/journal.pone.0154765)
Supplement: S1 File — (PDF) [file pone.0154765.s005.pdf]

Directed Chemical Evolution with an Outsized Genetic Code

Casey J. Krusemark, Nicolas P. Tilmans, Patrick O. Brown, Pehr B. Harbury

SUPPORTING EXPERIMENTAL PROCEDURES

Materials

Solvents and general chemistry reagents were purchased from VWR International (West Chester, PA), Fisher Scientific (Hampton, NH), or Sigma-Aldrich (St. Louis, MO) unless otherwise indicated. Fmoc protected amino acids were from Novabiochem (La Jolla, CA) or Chem-Impex (Wood Dale, IL). 5'-pentynyl oligonucleotides for anti-codon resin preparation and 40-mer oligonucleotides for genetic library assembly were purchased from Bioneer (Alameda, CA). All other oligonucleotides were purchased from Stanford PAN Facility (Stanford, CA). Primary amine functionalized oligonucleotide included Spacer Phosphoramidite 9 (10-1909-90) and 5'-amino-modifier TEG (10-1917-90) modifications from Glen Research (Sterling, VA). 384 well filter plates with 5.0 μm glass frits (EK-2287) used for anticodon resin preparation and for library chemistry were obtained from E&K Scientific (Santa Clara, CA). The catalytic subunit of murine cAMP-activated protein kinase A was from NEB (P6000L). The laser cutter was a Legend 36EXT from Epilog Laser (Golden, CO). The Kemptide PKA substrate peptide (100055-810) used for kinetic studies was obtained from AnaSpec (Fremont, CA).

Abbreviations

ATP (adenosine triphosphate), ATPγS (adenosine gamma-thio triphosphate), BSA (bovine serum albumin), cAMP (cyclic adenosine monophosphate), $CuSO_4$ (copper sulfate), DCE (dichloroethane), DCM (dichloromethane), DEAE (diethylaminoethyl), DIEA (diisopropylethylamine), DMF (dimethylformamide), DMSO (dimethylsulfoxide), dsDNA (double-stranded DNA), DTT (dithiothreitol), EDC (1-Ethyl-3-[3-dimethylaminopropyl] carbodiimide), EDTA (ethylenediaminetetraacetic acid), Fmoc (fluorenylmethoxycarbonyl), FPLC (fast protein liquid chromatography), H_2O (water), HOAt (1-Hydroxy-7-azabenzotriazole), LCMS (liquid chromatography mass spectrometry), $MgCl_2$ (magnesium chloride), MWCO (molecular weight cutoff), NaCl (sodium chloride), NaOAc (sodium acetate), NaOH (sodium hydroxide), NHS (N-hydroxysuccinimide), NTP's (nucleotide triphosphates), PEG (polyethylene glycol), PNK (polynucleotide kinase), PCR (polymerase chain reaction), qPCR (quantitative PCR), SDS (sodium dodecylsulfate), SSC (saline sodium citrate buffer), ssDNA (single-stranded DNA), TAE (tris-acetate, EDTA), TBE (tris-borate, EDTA), TEG (triethyleneglycol), TFA (trifluoroacetic acid), THPTA (tris[(1-hydroxy-propyl-1*H*-1,2,3-triazol-4-yl)methyl]amine), Tris (2-Amino-2-hydroxymethyl-propane-1,3-diol), tRNA (transfer ribonucleotide).

Selection for kinase substrates

Peptide-DNA conjugates for pilot selections

Oligonucleotide primers bearing a 5' Kemptide peptide, or the truncated Kemptide peptide RRASL, were prepared by covalent modification at the 5' end of the z_a primer: 5'-ATGGTATCAA GCTTGCCACA-3' as detailed below.

A protected Kemptide peptide coupled to chloro-trityl resin was purchased from the Stanford PAN facility. The crude resin (60 mg resin) was deprotected with a 1:1:2 solution of DMF:DCM:piperidine for 2.5 hours, and then washed with DMF and DCM. The peptide was then modified with an N-terminal PEG-azide moiety. To perform the modification, 11-Azido-3,6,9-trioxaundecan-1-amine (Sigma-Aldrich, 17758), was converted into an activated isocyanate as follows. The azide-PEG-amine (500 μmol) was mixed with DIEA (1.25 mmol) in 1 ml of DCE. This solution was added dropwise to a 1 ml triphosgene (166 μmol) solution in DCE and allowed to react for 30 min. The crude azide-PEG-isocyanate was mixed with peptide resin and allowed to react for 1.5 hours while tumbling at room temperature. The resin was washed in DCE and DCM and then dried overnight under vacuum. The peptide was cleaved from resin with 90% TFA, 4.5% DCE, 4.5% H_2O, 1% triisopropylsilane for 2 hours and precipitated with ether. Separately, an alkyne-z_a oligonucleotide was prepared by coupling 4-pentynoic acid (Sigma-Aldrich, 232211) to a z_a oligonucleotide with the C12 5'-amino-modifier (Glen Research, #10-1912). This coupling was performed in methanol on a DEAE Sepharose solid support using previously published procedures(1). The alkyne-modified oligonucleotide was purified by HPLC and ethanol precipitated. Cu^I-catalyzed azide-alkyne cycloaddition chemistry was used to link Kemptide to the oligonucleotide. The reaction consisted of 1.8 nmol alkyne- C12-z_a oligonucleotide, 50 nmol azide-PEG-Kemptide, 40 mM phosphate buffer pH 7.0, 5 mM aminoguanidine, 5 mM sodium ascorbate, 0.25 mM $CuSO_4$, 1.25 mM THPTA, in a 100 μl volume(2). The reaction proceeded for 2 hours at room temperature and was stopped by the addition of excess EDTA. The Kemptide-z_a conjugate was purified by HPLC. The identity of the product was confirmed by LC/MS ($[M+H]^+$, expected 1610.8, found 1611.5) after P1 Nuclease digestion of the oligonucleotide, as previously described(1).

A RRASL-z_a conjugate was prepared by direct Fmoc peptide synthesis on a DEAE-Sepharose solid support following previously published procedures(1). The z_a amino oligonucleotide had been modified with Spacer Phosphoramidite 9 and 5'-amino-modifier TEG (Glen Research, #10-1909-90 and #10-1917-90). The final arginine coupling was repeated three times, as Arg-Arg couplings proceed with low efficiency. The conjugate was eluted from DEAE and HPLC purified. The product was confirmed by LC/MS after P1 digestion ($[M+H]^+$, expected 1302.6, found 1302.7). The product was roughly a 2:1 mixture of the 1 and 2 arginine containing products (assuming equal ionization), which did not resolve on HPLC. The conjugate was used without further purification.

180 base-pair peptide-DNA conjugates were prepared by PCR using both the Kemptide-z_a and RRASL-z_a primers and the z_e' reverse primer(3). The dsDNA consisted of z_a-z_e with VA_{001}, VB_{001}, VC_{001} and VD_{001} at the variable positions. An heterologous unconjugated DNA of 180 bp length was

prepared by PCR from the plasmid pDEST-C102-Trx (a generous gift from Dr. Michael Dyson(4)) with the following primers: 5'-CAGCGCTACC TTGTCATTCA-3' and 5'-GTACCTTCGT GCACCACTT-3'.

Pilot selections

For pilot selections, one of the peptide-DNA conjugates was mixed with unconjugated DNA in an approximate ratio of 1 part in 10,000. 20 pmol of total input DNA was used for the Kemptide-DNA pilot selection and 100 pmol of total input DNA was used for the RRASL-DNA pilot selection. DNA was quantified by UV absorbance at 260 nm. The selections were performed as described in the main text. Enrichment was quantified by standard-curve qPCR on the eluted Miltenyi beads. qPCR was performed using SYBR Green PCR Master Mix (Applied Biosystems, 4309155) on an Applied Biosystems 7900HT Fast Real-Time PCR System. To quantify the substrate-DNA conjugate, the z_a primer and B001' (5'-CGGGCCTATG TACGCTAATC-3') were used at 0.5 μM concentration, which generated an 80 base-pair product. To quantify the unconjugated DNA, qPCR was performed with two heterologous primers (5'-CAGCGCTACC TTGTCATTCA-3' and 5'-CGTCGTCCAT GTCTTCTTCA-3') at 0.5 μM concentration, which generated a 103 base-pair product.

Selections applied to chemically-translated libraries

The selections were performed as described in the main text. The eluted post-selection Miltenyi beads were used as template in a 400 μl PCR reaction with 1 μM z_a and z_f' primers, 250 μM dNTPs, 1x DreamTaq buffer, and 10 units of DreamTaq DNA polymerase. A mixture of 12.5 pmols of the kinase-selected amplicons and 12.5 pmols of the mock-selected amplicons was used as starting material to generate ssDNA for the next round of DNA-programmed library synthesis. E-codon specific primers were used for the post-selection PCR amplification in the final selection round. The primers consisted of z_f' fused at the 3' end to a portion of VE_{001}' (5'-CTAGTACTCG AGATTCTGCC CGTCG-3') or to a portion of VE_{002}' (5'-CTAGTACTCG AGATTCTGCC GAGAC-3'). The E-codon specific primers supressed amplification of genes that had been sorted into the wrong selection pool (i.e. VE_{002} into the PKA-treated pool or VE_{001} into the mock-treated pool).

Reagents for DNA-programmed chemistry

Hybridization array preparation

Anticodon resin was prepared by Cu^{I}-catalyzed azide-alkyne cycloaddition of 5'-alkyne modified 20-mer oligonucleotides to azido-Sepharose. Azido-Sepharose was prepared from NHS-sepharose as previously described(5) or from CM-Sepharose. For preparation of azido-sepharose from CM-Sepharose, the resin was washed with H_2O and then with anhydrous DMF. The washed resin (5 ml settled volume) was activated with EDC (288 mg, 1.5 mmol) and HOAt (30 mg, 0.22 mmol) in minimal additional DMF. Azido-PEG400-amine (1.64 g, 3.75 mmol) was added and allowed to react overnight, tumbling at room temperature. The azide-modified resin was washed thoroughly with DMF, methanol and H_2O. Azido-Sepharose was stored at 5 °C in 40% ethanol.

To generate 384 distinct hybridization resins, 15 μl azido-sepharose was placed in each well of a 384-well filter plate and washed 3 times with 100 μl water. The wells of the filter plate were

stoppered with a rubber matt (silicone rubber 60 A durometer 1/4 inch thickness, McMaster-Carr, #8632K462) that had been laser cut to produce small holes centered on each feature. Click reactions contained 22.5 µM alkyne oligonucleotide (75 nmol/ml loading level), 75 µM phosphate buffer pH 7.0, 0.25 mM $CuSO_4$, 1.25 mM THPTA, 5 mM aminoguanidine, and 5 mM sodium ascorbate, in a 50 µl volume(2). Reactions proceeded for 1 hour at room temperature. The resin was washed 3 times with 85 µl of 60 mM phosphate pH 6.5, 1.5 M NaCl, 10 mM EDTA, 0.005% Triton X-100, followed by 3 washes with H_2O. The resin was stored in hybridization resin storage buffer (50 mM Tris pH 7.5, 100 mM NaCl, 1 mM EDTA, 0.05% sodium azide, 0.005% Triton X-100) at 5 °C and later transferred to an anticodon array.

For the larger scale VE anticodon columns, 150 µl portions of azido-Sepharose were washed with water in a 1.5 ml microfuge tube. Click reactions were performed as before in 500 µl volume and were tumbled at room temperature for 1 hour. The completed reactions were allowed to settle and decanted. 150 µl of 60 mM phosphate pH 6.5, 1.5 M NaCl, 10 mM EDTA, 0.005% Triton X-100 buffer was added to each tube, and the resin slurries were transferred to empty TWIST synthesis columns (#20–0030, Glen Research). The resins were washed in the column on a vacuum manifold, twice with 10 ml of high salt buffer and twice with 10 ml of H_2O. Each column was fitted with two 1 ml syringes, flushed with 6x SSC and held at 50 °C overnight. The columns were washed as before with high salt buffer and H_2O and then stored at 5 °C in hybridization storage buffer.

Anticodon arrays were prepared by previously-published procedures with slight modifications(5). Black acetal plastic (McMaster-Carr, 8492K213) of 1/16" thickness was abraded on the benchtop and then coated on both sides with double-sided silicone tape (Champion Tape D5250, Sturtevant, WI). A 16 by 24 array of 3.2 mm squares on 4.5 mm centers was laser cut into the acetal. The tape liner was removed from one side and a nylon netting (30 µm pore size, Small Parts B000FN0OG2) was applied. The open array was affixed with tape to the bottom half of a previously described chemistry device(5), including the bottom gasket, and placed on a vacuum manifold. 15 µl of prepared anticodon resin was pipetted into each 3.2 mm square reservoir. This was accomplished in several portions from a 1:3 resin:buffer susupension. Excess buffer was removed by vacuum. After filling, the array was removed from the chemistry device. The remaining tape liner was removed, and the 384 reservoirs were sealed with another sheet of nylon netting. To prevent the nylon netting from detaching during repeated use, the array was bonded on both sides with an adhesive polypropylene layer. Polypropylene sheeting (Celgard 2500, 25 µm thickness; Celgard, Charlotte, NC), or corona treated biaxially oriented polypropylene, 2 mil thickness (Griff Paper and Film, Fallsington, PA) was affixed to double-sided tape, and laser cut to create a 16 by 24 pattern of 3.2 mm squares on 4.5 mm centers. The adhesive-backed and laser-cut sheets were then applied to both nylon surfaces of a virgin array. After assembly, the arrays were washed with 50 ml of 6x SSC in a plastic bag on a shaker at 130 rpm at 50 °C overnight. The arrays were stored in hybridization storage buffer at 5 °C.

Gene library assembly

The general structure of the DNA library was similar to previous work(5). Briefly, the library consisted of six 20-base noncoding regions (z_a-z_f) of constant sequence alternating with 5 variable, 20-base coding regions (designated VA, VB, VC, VD and VE) to give 220 base-pair genes. At each of the VA-VD coding regions, 384 mutually exclusive sequences were present for a total of 1536 different 20-mers ($VA_{001-384}$, $VB_{001-384}$, $VC_{001-384}$, $VD_{001-384}$), using previously reported sequences(3). The fifth 20-base coding region contained either of two sequences (VE_{001} or VE_{002}) for a total of 4.3 x 10^{10} (384^4 x 2) different genes. The VE coding region was used to split the library into mock-selected and kinase selected pools.

The library was assembled from 40-mer oligonucleotides. Each oligonucleotide consisted of a constant region (one of z_a-z_f or their complements) at the 5' end and a coding region (one of $VA_{001-384}$, $VB_{001-384}$, $VC_{001-384}$, $VD_{001-384}$ or their complements) at the 3' end. Sets of 96 different 40-mers, containing 48 pairs with complementary 3'-ends, were combined into pools. There were 8 pools for each coding region. The pooled oligos were diluted to 4.5 µM concentration in 1x T4 PNK buffer (Fermentas), denatured at high temperature, and then slowly cooled in a thermocycler to allow hybridization (program: 95 °C 5 min, 85 °C 1 min, drop 1 °C per min for 26 min, 58 °C 5 min, drop 1 °C per 0.5 min for 15 min). The 8 pools of hybridized 40-mers for each coding region were combined and phosphorylated with T4 PNK (Fermentas, EK0031). Reactions contained 3.2 µM oligonucleotide, 1 mM ATP, 1x PNK buffer, 0.11 units/µl PNK in 750 µl volume and proceeded for 8 hours at 37 °C. The reactions were concentrated to 250 µl by n-butanol extractions and ethanol precipitated in the presence of 10 mM $MgCl_2$. The pellets were resuspended in 200 µl TAE and the oligonucleotide dimers were purified by agarose gel electrophoresis to remove any non-hybridized monomer oligonucleotides. The dimers were recovered by electroelution. Excised gel slices were placed in dialysis bags (10K MWCO) with 1 ml TAE buffer and electrophoresed in an Owl gel box for 30 min at a constant 125 V. Recovered DNA was ethanol precipitated as before and quantified. 15 µg (~0.63 nmol) portions of DNA from each coding region were combined and ligated together. Ligation reactions contained 5% PEG 4000, 1x T4 ligation buffer, 0.1 units/µl DNA ligase in a 500 µl solution and proceeded overnight at 37 °C. The reaction was phenol/chloroform extracted, n-butanol concentrated to 300 µl, and isopropanol precipitated. Fully assembled library was purified on a 5% acrylamide TBE formamide(40%)-urea (7 M) denaturing gel. The gel was run at 15 watts constant power for 50 minutes. Full length 220 base-pair genes from excised gel slices were recovered by electroelution as described above. A 10 pmol aliquot of the gel-purified genes was PCR amplified with z_a and z_f' primers to produce the zeroeth-generation library material.

Preparation of ssDNA and library diversification

A 25 pmol portion of library template was PCR amplified with primers that appended a T7 RNA polymerase promoter. The sequence of the primer (T7-z_f') was: 5'-GCGCTTAATA CGACTCACTA TAGGGAGACT AGTACTCGAG ATTCTGCC-3'. A 60 pmol portion of the PCR product was used as a template in an *in vitro* transcription reaction to generate RNA. The reaction contained 1x nucleic acid sequence-based amplification buffer(6) (37 mM Tris pH 8.5, 18.5 mM $MgCl_2$, 37 mM KCl, 9.3% DMSO), 5 mM DTT, 0.1 mg/ml BSA, 3.5 mM NTPs, 0.6 U/µl T7 RNA Polymerase,

0.5 U/μl pyrophosphatase, and 250 nM DNA template. The reaction proceeded for 6 hours at 42 °C. RNA was isolated using four PureLink RNA Mini kit columns (Invitrogen) according to the manufacturer's instructions. The DNA template was removed by on-column DNase I digestion. 20 nmol of the RNA product was then used as template in a reverse transcription reaction to generate RNA:DNA heteroduplexes. Reaction conditions were modified slightly from the manufacturer's protocol. The reaction contained 0.2 μg/μl (3.0 μM) RNA template, 3 μM 5'-amine oligonucleotide primer or 5'-^{32}P labeled primer, 1 mM dNTPs, 1x FirstStrand buffer, 5 mM DTT, 1 U/μl superscript III reverse transcriptase (Invitrogen), 0.0005 U/μl pyrophosphatase, and 0.2 U/μl RiboLock ribonuclease inhibitor (Fermentas). Following incubation at 50 °C for 4 hours, the product was subjected twice to a 1:1 phenol:chloroform extraction. The solution was concentrated to 800 μl by repeated extractions with n-butanol. Heteroduplex nucleic acid was precipitated by the addition of a half volume of 7.5 M ammonium acetate and 2 volumes of isopropanol. After centrifugation at ~14,000 g for 30 min at 4 °C, the pellet was washed thoroughly with 80% ethanol and allowed to air dry. The recovered nucleic acid was dissolved in 200 μl H_2O and quantified. The solubilized pellet was combined with 20-mer oligonucleotides complementary to the constant regions (10 μM each) and diluted to approximately 2 ml into 100 mM NaOH. The solution was heated to 100 °C for 5 min to hydrolyse the RNA. Following neutralization with 1 M acetic acid, the reaction was diluted to a 3 ml final volume with 5 mM Tris pH 7.5, 1 mM EDTA, 0.5x SSC, 0.5 mg/ml BSA, and 0.5 mg/ml yeast tRNA. For the fourth round of chemical translation, the final salt concentration was raised to 1.5x SSC and SDS was added to 0.02%.

Library diversification was accomplished by gene recombination during ssDNA preparation. Recombinants were produced by template switching at the constant regions during the transcription, reverse transcription and replication steps. To quantify the recombination rate, we prepared ssDNA from pure single genes (the 001 dsDNA template used for pilot selections, and an 002 dsDNA template consisting of z_a-z_e with VA_{002}, VB_{002}, VC_{002} and VD_{002} at the variable positions). In parallel reactions, ssDNA was generated from the 001 template, the 002 template, and from an equal mixture of the two. The fraction of recombinants in each preparation was quantified by standard-curve qPCR. A mixture of the pure 001 and pure 002 products (mixed just prior to qPCR) served as a negative control. 2-5% of the genes in the mixed template ssDNA preparation were recombinants of the parent genes. Less than 0.1% of the genes in the negative control were recombinants.

DNA-programmed chemistry

DNA hybridization, transfer, and chemistry

The ssDNA library was applied to each anticodon array using a previously described hybridization pump(5) with slight modifications. Prior to application of DNA to the array, the array and pump were equilibrated in hybridization buffer (translations 1-3: 1x SSC with 5 mM Tris pH 7.5, 1 mM EDTA, 0.005% Triton X-100; translation 4: 2x SSC with 10 mM Tris pH 7.5, 1 mM EDTA, 0.005% Triton X-100, 0.02% SDS). The ssDNA library was then pumped through the array overnight at 37 °C.

For translations 1-3, the array was washed twice with 15 ml portions of 1x SSC in the hybridization pump. For translation 4 only, the array was washed once in the pump with 15 ml 2x SSC (with no SDS) and then washed in plastic bags on a 130 rpm shaker at 37 °C for 15 min in 50 ml 10x SSC and then twice with 50 ml of 0.25x SSC. The arrays were patted dry and imaged with a phosphor-imaging screen. The arrays were then mounted into a previously described chemistry device(5), which uses rubber gaskets to form an isolated liquid channel above and below each feature. The mounted array was washed twice with 50 ml portions of 0.25x SSC on a vacuum manifold and then centrifuged at 1000 rpm for 1 min to remove residual buffer.

To elute DNA from the anticodon array, the mounted array was placed on top of a vacuum manifold and 35 µl of DNA-denaturing buffer (10 mM NaOH with 1 mM EDTA and 0.005% Triton X-100) was applied to each well. For the fourth round, this volume was reduced to 30 µl. Care was taken to ensure that the resin in each feature absorbed the entire volume and that liquid did not leak from the bottom. The array was incubated at room temperature for 10 min. Separately, 5 µl of DEAE-Sepharose was pipetted from a 50:50 slurry into each well of a 384-well filter plate. The resin was washed twice with 85 µl H_2O and twice with 10 mM acetic acid with 0.005% Triton X-100. Then 15 µl of 60 mM acetic acid was added to each well, and the filter plate was sealed on the bottom with a rubber stopper. After incubation of the anticodon array in the DNA-denaturing buffer, the mounted array was placed on top of the stoppered filter plate, taking care to maintain registration of the 384 features. The entire stack was centrifuged at 1000 rpm for 1 min. The anticodon array in the device was removed and incubated as before with DNA-denaturing buffer. The filter plate was sealed on top with packing tape. The plate was affixed to the top of a Thermomixer (Eppendorf) and shaken for 10 min at 1200 rpm. The packing tape and rubber stopper were removed from the filter plate, and the filtrate was eliminated by centrifugation or vacuum. As before, 15 µl of 60 mM acetic acid was added to each well and a second transfer step was performed. The entire procedure was repeated a third time to complete the transfer of DNA.

The DEAE resin in the filter plate was washed three times with 85 µl H_2O and then three times with 85 µl dry methanol. Peptide couplings were performed as previously described using EDC and HOAt in methanol and DMF(1). The couplings proceeded for 20 minutes, after which the wells were then washed twice with 85 µl of methanol and twice with 85 µl of DMF. Fmoc deprotection was carried out by a 20-minute incubation with 20% piperidine in DMF. The wells were then washed twice with 85 µl of DMF. For translations 1-3, the couplings were performed only once. For the fourth translation, double couplings were performed, and an additional 3 couplings with Fmoc-arginine were performed at features encoding an arginine dimer. Each amino acid was coupled to 12 different array positions during the first two translations. This was reduced to a subset of 6 array positions for the last two translations.

Following the chemistry, the DNA was eluted from the DEAE-Sepharose resin with two 50 µl aliquots of 50 mM NaOH, 1.5 M NaCl, 1 mM EDTA, and 0.005% Triton X-100. During the elution steps, the filter plate was stoppered, covered with tape, and shaken on the Thermomixer for 15 min at 1200 rpm. Then, the filtrate was collected by centrifugation into a polypropylene 384-well plate. The

two filtrate fractions were pooled and neutralized by the addition of 1 M Tris pH 7.5 to 75 mM final concentration. The solution was concentrated and buffer exchanged into hybridization buffer with a 10,000 Da molecular weight cut-off centrifugal filter device (GE Healthcare). BSA and tRNA were added to 0.5 mg/ml each. The sample was diluted to 3 ml with hybridization buffer and applied to the next anticodon array for additional chemical steps, or was applied to E-region anticodon columns for subsequent selection steps.

Library splitting into mock and kinase-selected pools

The VE_{001} and VE_{002} anticodon columns were equilibrated in hybridization buffer with a peristaltic pump. DNA was pumped cyclically through the columns at maximum speed for 3 hours at 37 °C. The columns were then washed with 15 ml of hybridization buffer. For the final chemical translation, the columns were additionally washed with 15 ml of 10x SSC and then 15 ml of 0.25x SSC. DNA was eluted from the anticodon columns by two 750 µl aliquots of DNA-denaturing buffer, each applied at 37 °C for 15 minutes. The eluted fractions were pooled and neutralized by addition of Tris pH 7.5 to a final concentration of 75 mM. The DNA was concentrated with n-butanol and isopropanol precipitated. The ssDNA pellets were dissolved in 200 µl H_2O and duplexed to dsDNA with single-cycle PCR reactions (95 °C for 2.5 min, 58 °C for 1 min, 72 °C for 15 min). The reactions contained 1x DreamTaq buffer, 250 µM dNTPs, 4 µM z_f' 20-mer oligonucleotide, and 20 units of DreamTaq DNA polymerase in a 400 µl volume. Following duplex formation, the DNA was phenol:$CHCl_3$ extracted, concentrated with n-butanol and isopropanol precipitated.

Illumina sequencing

25 pmol of amplified DNA from the initial assembled library (generation 0) and from generations 2, 3, and 4 selected material were used as template in PCR reactions that append Illumina adaptor sequences. The two PCR primers had the following sequences: 5'-AATGATACGG CGACCACCGA GATCTACACT ATGGTATCAA GCTTGCCACA-3', and 5'-CAAGCAGAAG ACGGCATACG AGATCGTACT AGTACTCGAG ATTCTGCC-3'. This PCR product was quantified with an Agilent 2100 Bioanalyzer, and a 10 nM solution was submitted for sequencing by paired-end, 150 base-pair reads on a MiSeq Illumina Sequencer. The following custom sequencing primers were used: 5'-CACCGAGATC TACACTATGG TATCAAGCTT GCCACA-3' (read 1), and 5'-CGGCATACGA GATCGTACTA GTACTCGAGA TTCTGCC-3' (read 2). DNA sequencing was performed at the Stanford Functional Genomics Facility.

SUPPLEMENTAL REFERENCES

1. Halpin, D.R., Lee, J.A., Wrenn, S.J. and Harbury, P.B. (2004) DNA display III. Solid-phase organic synthesis on unprotected DNA. *PLoS Biology*, **2**, E175.

2. Hong, V., Presolski, S.I., Ma, C. and Finn, M.G. (2009) Analysis and Optimization of Copper-Catalyzed Azide–Alkyne Cycloaddition for Bioconjugation. *Angewandte Chemie International Edition*, **48**, 9879-9883.

3. Weisinger, R.M., Wrenn, S.J. and Harbury, P.B. (2012) Highly parallel translation of DNA sequences into small molecules. *PLoS ONE*, **7**, e28056.

4. Dyson, M.R., Shadbolt, S.P., Vincent, K.J., Perera, R.L. and McCafferty, J. (2004) Production of soluble mammalian proteins in Escherichia coli: identification of protein features that correlate with successful expression. *BMC Biotechnol*, **4**, 32.

5. Weisinger, R.M., Marinelli, R.J., Wrenn, S.J. and Harbury, P.B. (2012) Mesofluidic Devices for DNA-Programmed Combinatorial Chemistry. *PLoS ONE*, **7**, e32299.

6. Strijp, D. and Aarle, P. (1998), *T Diagnostic Virology Protocols*, Vol. 12, pp. 331-340.
